# Effect of TSH on oocyte maturation of PCOS patients with normal thyroid function in IVF

**DOI:** 10.1186/s12958-022-01005-1

**Published:** 2022-09-02

**Authors:** Shaoyuan Xu, Ying Zhang, Cancan Qiang, Changjun Zhang

**Affiliations:** 1 Department of Human Reproductive Center, Renmin Hospital, Hubei University of Medicine, Shiyan, Hubei China; 2 Hubei Clinical Research Center for Reproductive Medicine, Shiyan, Hubei China; 3 Department of Cardiology, Renmin Hospital, Hubei University of Medicine, Shiyan, China

**Keywords:** Fertilization, In vitro, PCOS, TSH, Oocyte maturation, Pregnancy

## Abstract

**Background:**

The serum TSH level of PCOS patients was higher than that of the general female population. For patients with thyroid dysfunction, the abnormal TSH level is negatively related to the outcomes of assisted reproductive technology, but for PCOS patients with normal thyroid function, the effect of TSH level on outcomes of in vitro fertilization has not been reported. In this study, PCOS patients with normal thyroid function were included in this study to evaluate the effect of TSH on the outcomes of IVF-ET.

**Methods:**

A retrospective cohort study was conducted to analyze the clinical data of 3190 patients who underwent IVF-ET in the Department of Human Reproductive Center of Renmin Hospital Hubei University of Medicine from January 2017 to July 2021, including 594 PCOS patients and 2595 non PCOS patients. The IVF-ET outcomes between the two groups were compared; Multi-factor linear regression analysis was used to analyze the correlation between the related variables and the oocyte maturation of PCOS patients; The ROC curve of the effect of TSH on oocyte maturation in PCOS patients was drawn. The PCOS patients were divided into TSH < 2.98 group (*n* = 454) and TSH ≥ 2.98 group (*n* = 141) according to ROC threshold TSH 2.98, and the outcomes were compared.

**Results:**

TSH level in PCOS group was significantly higher than that in non-PCOS group (2.42 ± 0.86 vs 2.00 ± 0.89 UU / ml, *P* < 0.01), and the oocyte maturation rate and 2PN fertilization rate in PCOS group were lower than those in non-PCOS group (90.9% vs 92.4%, *P* = 0.02) (84.57% vs 86.77%, *P* = 0.02). There was no significant difference in cleavage rate and high-quality embryo rate between the two groups (*P* > 0.05); There was no difference in clinical pregnancy rate, abortion rate, ectopic pregnancy rate and live birth rate between the two groups (*P* > 0.05).

Multi-factor linear regression analysis showed that TSH was negatively correlated with oocyte maturation in PCOS patients [β = -0.124, *P* = 0.013,95%CI (-0.027 ~ -0.003)]; The oocyte maturation rate in TSH < 2.98 group was significantly higher than that in TSH ≥ 2.98 group (91.7% vs 88.2%, *P* = 0.001).

**Conclusion:**

The TSH level of PCOS patients with normal thyroid function is higher than that of normal people, and it is negatively correlated with the oocyte maturation in in-vitro fertilization. The ROC curve showed that when TSH > 2.98uIU/ml, the possibility of immature oocytes was higher (specificity 28.9%, sensitivity 83.0%).

## Introduction

Polycystic ovary syndrome (PCOS) is a kind of endocrine and metabolic disorder disease, which is common in women of childbearing age, with a incidence rate of 15%, and it’s main clinical manifestations are rare ovulation or anovulation, accompanied by abnormal glucose and lipid metabolism and hyperandrogenemia [1, 2].The pathogenesis of PCOS is complex, at present it is mainly believed that the pathogenesis of PCOS is related to environmental, genetic, immune and endocrine regulation [3]. In vitro fertilization embryo transfer (IVF-ET) is an effective method to treat infertility in PCOS patients [4].

The hypothalamus pituitary ovary axis and hypothalamus pituitary thyroid axis interact and influence each other. Abnormal thyroid stimulating hormone (TSH) level can affect female metabolism and reproductive system function [5–7]. At the same time, the gonadal axis imbalance caused by endocrine hormone disorders in PCOS patients also affects the pituitary thyroid axis, the serum TSH level of PCOS patients was higher than that of the general female population [8–10].Thyroid stimulating hormone(TSH)is the most sensitive and commonly used index to evaluate thyroid function [11, 12]. Both The American Society for reproductive medicine (ASRM) and The American Thyroid Association (ATA) recommended routine thyroid function test for pregnant women [13, 14]. For patients with thyroid dysfunction, it is widely acknowledged that abnormal TSH level is related to the decrease of clinical pregnancy rate and increase of abortion rate in assisted reproductive technology [15–17], but for PCOS patients with normal thyroid function, the effect of TSH level on outcomes of IVF-ET has not been rarely reported.

In this study, PCOS patients with normal thyroid function were included in this study to evaluate the effect of TSH on the outcomes of IVF-ET.

## Materials and methods

### Participants

A retrospective cohort study was conducted to analyze the clinical data of 3190 patients who underwent IVF-ET in the Department of Human Reproductive Center of Renmin Hospital Hubei University of Medicine from January 2017 to July 2021.

#### Inclusion criteria

①Age < 40,②AMH > 1 ng/ml,③The TSH level measured on the third day of menstruation was in the normal range of our laboratory (0.56–5.91uIU/ml).

#### Exclusion criteria

①Previous history of thyroid disease,②High risk factors of recurrent abortion,③Patients with underlying diseases who are not suitable for pregnancy.

Three thousand one hundred ninety patients were divided into two groups, including 594 patients in PCOS group and 2595 patients in non PCOS group. According to the threshold TSH2.98 of ROC curve, all the PCOS patients were divided into two groups: TSH < 2.98 group (*n* = 454) and TSH ≥ 2.98 group (*n* = 141). Rotterdam criteria [1] were used for the diagnosis of PCOS.

This study was approved by the Ethics Committee of Renmin Hospital, Hubei University of Medicine and conducted in conformity with the Helsinki Declaration.

### Ovulation induction protocol

All patients were treated with GnRH-agonist long protocol. Triptorelin 3.75 mg (Germany Huiling Pharmaceutical Co., Ltd.) was injected on the third day of menstruation. After 30 days, gonadotropins (r-FSH, Gonafen, Merck Serono, or HMG, Livzon Medicine) were used for ovulation induction. When multiple follicles matured (the diameter of follicles measured by ultrasound was over 18 mm), 250 μg of recombinant human chorionic gonadotropin (r-HCG, Merck serono) was injected, 36 h later B-ultrasound guided transvaginal aspiration was performed to evaluate the quality of oocytes retrieved. According to the maturation, the oocytes retrieved were divided into three stages: germinal vesicle phase (GV), meiosis metaphase I (M I), meiosis metaphase II (M II), and M II was mature oocytes.

### Embryo culture, corpus luteum support and diagnosis of clinical pregnancy

The oocytes retrieved were subjected to embryo culture after conventional IVF fertilization or ICSI fertilization. 16 ~ 18 h after fertilization, the pronucleus were observed, and the appearance of two pronucleus was judged as normal fertilization. The embryo scoring method referred to Ziebe etc. [18]. Grade I ~ II are high-quality embryos; Grade I ~ III is usable embryo; Grade IV embryos are discarded embryos. High quality embryo rate = (Grade I + Grade II) embryos/total number of embryos, and available embryo rate = (Grade I + Grade II + Grade III)/total number of embryos. From the day of oocytes retrieving, 90 mg/d of vaginal progesterone vaginal sustained-release gel (Merck Serono, Switzerland) and 20 mg/d of oral dydrogesterone (Solvay Pharma, Netherlands) were used for luteal support. 14 days after embryo transfer, the positive serum HCG was judged as biochemical pregnancy, 30 days after embryo transfer, the original fetal heart beat was detected by B-ultrasound, which was judged as clinical pregnancy, the luteal support medication was continued until 60 days after embryo transfer. Pregnancy loss occurring before 12 weeks of gestation is judged as early spontaneous abortion.

### Observation indicators

ART cycle characteristics and embryologic outcomes**:** Oocyte maturation rate (number of metaphase II (MII) stage oocytes/number of oocytes retrieved × 100%), 2PN fertilization rate (2 pronuclei number/number of oocytes retrieved × 100%), High quality embryo rate (number of high-quality embryos/normal cleavage number × 100%).

Pregnancy outcomes: clinical pregnancy rate (number of pregnancy cycles/number of transplantation cycles × 100%), early spontaneous abortion rate (number of abortion cycles before 12 weeks/number of pregnancy cycles × 100%), live birth rate (number of births of live babies/number of total transplantation cycles × 100%).

### Statistical method

SPSS22.0 software was used for statistical analysis. The measurement data conform to the normal distribution expressed by the mean standard deviation (Mean ± SD), and the comparison between groups was made by one-way ANOVA. The counting data is expressed by the rate (%), and the comparison between groups adopts χ2 test; multi-factor linear regression analysis was used to exclude the influence of confounding factor and analyze the related variables affecting the oocyte maturation of PCOS patients. The ROC curve of the effect of TSH on oocyte maturation of PCOS patients was drawn. *P* < 0.05 was statistically significant.

## Result

### Comparison of Baseline characteristics of PCOS group and non-PCOS group (see Table 1 for details)

**Table 1:** Characteristic of PCOS group and non-PCOS group

Characteristic	PCOS Group (*n* = 595)	Non-PCOS Group (*n* = 2595)	t/χ2 value	*P* value
TSH (uIU/ml)	2.42 ± 0.86	2.00 ± 0.89	107.79	< 0.001***
Age (year)	30.58 ± 3.03	30.81 ± 3.35	2.35	0.125
Infertility duration (year)	3.41 ± 2.24	3.42 ± 2.46	0.006	0.937
AMH (ng/ml)	10.58 ± 3.972	4.32 ± 1.794	3144.14	< 0.001***
Basal serum FSH (IU/L)	6.27 ± 1.48	6.20 ± 1.88	0.74	0.388
Basal serum LH (IU/L)	9.77 ± 10.68	6.88 ± 5.66	81.15	< 0.001***
Basal serum E2 (pg/ml)	44.26 ± 19.17	45.53 ± 20.91	1.58	0.209
Basal serum P (ng/ml)	0.58 ± 0.38	0.59 ± 0.39	0.22	0.639
Basal serum T (ng/ml)	0.60 ± 0.25	0.51 ± 0.22	76.78	< 0.001***
Basal serum PRL (ng/ml)	11.91 ± 6.08	13.24 ± 6.39	20.80	< 0.001***
Antral Follicle Counting(AFC))	12.18 ± 3.98	7.69 ± 2.54	1078.91	< 0.001***
Height (cm)	159.01 ± 5.42	158.89 ± 5.34	0.25	0.616
Weight (kg)	61.44 ± 10.15	58.56 ± 10.11	39.24	< 0.001***
Body Mass Index (kg/m2)	24.30 ± 3.93	23.18 ± 3.77	41.64	< 0.001***

^*^P < 0.05

^**^P < 0.01

^***^P < 0.001 for t test

There were significant differences in AMH, AFC, body weight, BMI, basal endocrine LH, T, PRL between the PCOS group and non-PCOS group (*P* < 0.001). TSH in PCOS group was significantly higher than that in non-PCOS group (2.42 ± 0.86 vs 2.00 ± 0.89 uIU/ml, *P* < 0.001).

### Comparison of embryologic outcomes and pregnancy outcomes of PCOS group and non-PCOS group (see Table 2 for details)

**Table 2:** Embryologic outcomes and pregnancy outcomes of PCOS group and non-PCOS group

Outcomes	PCOS Group (*n* = 595)	Non-PCOS Group (*n* = 2595)	t/χ2 value	*P* value
Gn used dosage (IU)	2268.67 ± 889.98	2564.74 ± 979.87	45.67	< 0.001***
Gn used duration (d)	11.57 ± 1.90	11.40 ± 2.46	2.46	0.116
FSH levels on HCG trigger day (IU/L)	13.46 ± 4.73	17.28 ± 6.19	193.59	< 0.001***
LH levels on HCG trigger day (IU/L)	1.06 ± 0.83	1.35 ± 1.09	35.59	< 0.001***
E2 levels on HCG trigger day (pg/ml)	2737.28 ± 1193.56	2625.42 ± 1152.41	4.39	0.036*
P levels on HCG trigger day (ng/ml)	0.75 ± 0.42	0.80 ± 0.38	8.74	0.003**
Endometrial thickness on HCG trigger day (mm)	11.71 ± 2.54	11.64 ± 2.59	0.31	0.573
Number of oocytes retrieved	11.08 ± 2.84	9.60 ± 2.61	150.26	< 0.001***
Number of MII stage oocytes	10.05 ± 2.76	8.89 ± 2.64	90.68	< 0.001***
Oocyte maturation rate (%)	90.7% (5980/6594)	92.6% (23,082/24917)	25.57	< 0.001***
2PN fertilization rate (%)	84.57%	86.77%	13.35	< 0.001***
Cleavage rate (%)	99.38%	99.30%	0.29	0.588
Number of useable embryos	4.20 ± 1.319	3.91 ± 1.233	26.90	< 0.001***
Number of high-quality embryos	3.16 ± 1.66	2.95 ± 1.58	8.83	0.003**
High-quality embryo rate (%)	70.6%	71.0%	0.13	0.716
Clinical pregnancy rate (%)	62.7% (373/595)	62.7% (1628/2595)	0	0.983
Ectopic pregnancy rate (%)	0.8% (3/373)	1.4% (23/1628)	0.87	0.349
Early abortion rate (%)	15.8% (59/373)	13.5% (220/1628)	1.34	0.247
Live birth rate (%)	52.3% (311/595)	53.3% (1388/2595)	0.28	0.591

^*^P < 0.05

^**^P < 0.01

^***^P < 0.001 for t test or Chi-square test

Compared with non-PCOS group, Gn used Dosage, Number of oocytes retrieved, Number of MII stage oocytes, Number of available embryos and Number of high-quality embryos in PCOS group were significantly higher than those in non-PCOS group. The oocyte maturation rate and 2PN fertilization rate in PCOS group were lower than those in non-PCOS group (90.9% vs 92.4%, *P* = 0.02) (84.57% vs 86.77%, *P* = 0.02), and there was no significant difference in cleavage rate and high-quality embryo rate between the two groups (*P* > 0.05); There was no difference in clinical pregnancy rate, early abortion rate, ectopic pregnancy rate and live birth rate between the two groups (*P* > 0.05).

### Linear regression analysis between related variables and oocyte maturation of each PCOS patient (see Table 3 for details)

**Table 3:** Linear regression analysis results of oocyte maturation-related factors

Variable	Regression coefficient B	Standard error	Standard regression coefficient β	t value	*P* value	95% confidence interval
TSH (uIU/ml)	-0.015	0.006	-0.124	-2.487	0.013	-0.027 ~ -0.003
Age (year)	0.002	0.002	0.068	1.263	0.208	-0.001 ~ 0.006
Infertility duration (year)	0.001	0.002	0.015	0.285	0.776	-0.004 ~ 0.006
AMH (ng/ml)	0.002	0.002	0.083	1.418	0.157	-0.001 ~ 0.005
Basal serum FSH (IU/L)	0.001	0.004	0.015	0.281	0.779	-0.007 ~ 0.010
Basal serum LH (IU/L)	0.000	0.000	0.043	0.828	0.408	-0.001 ~ 0.001
Basal serum E2 (pg/ml)	1.650E-5	0.000	0.003	0.056	0.955	-0.001 ~ 0.001
Basal serum P (ng/ml)	0.016	0.015	0.055	1.060	0.290	-0.013 ~ 0.044
Basal serum T (ng/ml)	-0.001	0.021	-0.002	-0.039	0.969	-0.042 ~ 0.041
Antral Follicle Counting(AFC))	0.001	0.002	0.029	0.511	0.610	-0.002 ~ 0.004
Body Mass Index (kg/m2)	0.002	0.002	0.081	1.281	0.201	-0.001 ~ 0.006
Gn used dosage (IU)	1.049E-5	0.000	0.087	0.764	0.445	0.000 ~ 0.000
Gn used duration (d)	0.003	0.005	0.048	0.568	0.570	-0.007 ~ 0.013
FSH levels on HCG trigger day (IU/L)	2.079E-5	0.002	0.001	0.011	0.991	-0.004 ~ 0.004
LH levels on HCG trigger day (IU/L)	3.135E-5	0.009	0.000	0.003	0.997	-0.018 ~ 0.018
E2 levels on HCG trigger day (pg/ml)	4.636E-6	0.000	0.048	0.910	0.364	0.000 ~ 0.000
P levels on HCG trigger day (ng/ml)	0.007	0.016	0.024	0.419	0.676	-0.025 ~ 0.038
Endometrial thickness on HCG trigger day (mm)	-0.002	0.002	-0.044	-0.874	0.383	-0.006 ~ 0.002

The multi-factor linear regression analysis was used to evaluates the correlation between related variables and oocyte maturation of each PCOS patients. After controlled for confounding factors: Age, Infertility duration, AMH, Basal serum FSH, LH, E2, P, T, AFC, BMI, Gn used dosage, Gn used duration, the results showed that TSH was negatively correlated with oocyte maturation of each PCOS patient [β = -0.124, *P* = 0.013,95%CI (-0.027 ~ -0.003)].

### ROC curve of effect of TSH on oocyte maturation of each PCOS patient and Comparison of TSH < 2.98 group and TSH ≥ 2.98 group (see Table 4 for details)

**Table 4:** Reproductive outcomes of TSH < 2.98 group and TSH ≥ 2.98 group

Parameter	TSH < 2.98 group (*n* = 454)	TSH ≥ 2.98 group (*n* = 141)	t/χ2 value	*P* value
TSH (uIU/ml)	2.06 ± 0.62	3.56 ± 0.41	716.78	< 0.001***
Age (year)	30.64 ± 3.09	30.41 ± 2.85	0.59	0.442
Infertility duration (year)	3.39 ± 2.29	3.47 ± 2.10	0.13	0.713
AMH (ng/ml)	10.58 ± 4.00	10.55 ± 3.89	0.009	0.926
Basal serum FSH (IU/L)	6.26 ± 1.46	6.33 ± 1.55	0.25	0.613
Basal serum LH (IU/L)	9.80 ± 11.60	9.66 ± 7.02	0.01	0.892
Basal serum E2 (pg/ml)	44.57 ± 18.79	43.31 ± 20.30	0.42	0.514
Basal serum P (ng/ml)	0.58 ± 0.39	0.57 ± 0.36	0.02	0.868
Basal serum T (ng/ml)	0.61 ± 0.25	0.59 ± 0.28	0.58	0.444
Basal serum PRL (ng/ml)	11.66 ± 6.24	12.69 ± 5.44	3.01	0.083
Antral Follicle Counting(AFC))	12.04 ± 3.98	12.65 ± 3.96	2.32	0.128
Height (cm)	159.04 ± 5.41	158.89 ± 5.47	0.08	0.769
Weight (kg)	61.39 ± 10.41	61.65 ± 9.27	0.06	0.792
Body Mass Index (kg/m2)	24.25 ± 3.99	24.45 ± 3.75	0.26	0.605
Gn used dosage (IU)	2278.91 ± 911.08	2235.69 ± 820.56	0.253	0.615
Gn used duration (d)	11.65 ± 1.91	11.31 ± 1.81	3.50	0.062
FSH levels on HCG trigger day (IU/L)	13.36 ± 4.80	13.80 ± 4.48	0.90	0.341
LH levels on HCG trigger day (IU/L)	1.06 ± 0.84	1.03 ± 0.82	0.14	0.708
E2 levels on HCG trigger day (pg/ml)	2747.72 ± 1157.98	2703.69 ± 1305.39	0.14	0.705
P levels on HCG trigger day (ng/ml)	0.75 ± 0.43	0.76 ± 0.38	0.04	0.825
Endometrial thickness on HCG trigger day (mm)	11.71 ± 2.61	11.71 ± 2.34	0.001	0.982
Number of oocytes retrieved	11.02 ± 2.75	11.28 ± 3.10	0.92	0.336
Number of MII stage oocytes	10.09 ± 2.72	9.91 ± 2.86	0.49	0.483
Oocyte maturation rate (%)	91.7%	88.2%	10.86	0.001**
2PN fertilization rate (%)	84.61%	84.48%	0.01	0.914
Cleavage rate (%)	99.51%	98.96%	3.73	0.054
Number of useable embryos	4.24 ± 1.31	4.06 ± 1.34	2.02	0.156
Number of high-quality embryos	3.21 ± 1.66	3.02 ± 1.69	1.33	0.249
High-quality embryo rate (%)	71.1%	69.0%	0.63	0.426
Clinical pregnancy rate (%)	64.3% (292/454)	57.4% (81/141)	2.17	0.085
Ectopic pregnancy rate (%)	1.0% (3/292)	0% (0/81)	0.83	0.479
Early abortion rate (%)	15.4% (45/292)	17.3% (14/81)	0.16	0.398
Live birth rate (%)	53.7% (244/454)	47.5% (67/141)	1.67	0.116

^*^P < 0.05

^**^P < 0.01

^***^P < 0.001 for t test or Chi-square test

According to the oocyte maturation of each patient, the ROC curve of TSH predicting oocyte maturation of each PCOS patient was drawn. The results showed that the area under the curve was 0.550, the cut-off value was TSH2.98uIU/ml, the specificity was 28.9%, the sensitivity was 83.0%, *P* = 0.037.

**Fig. 1:**
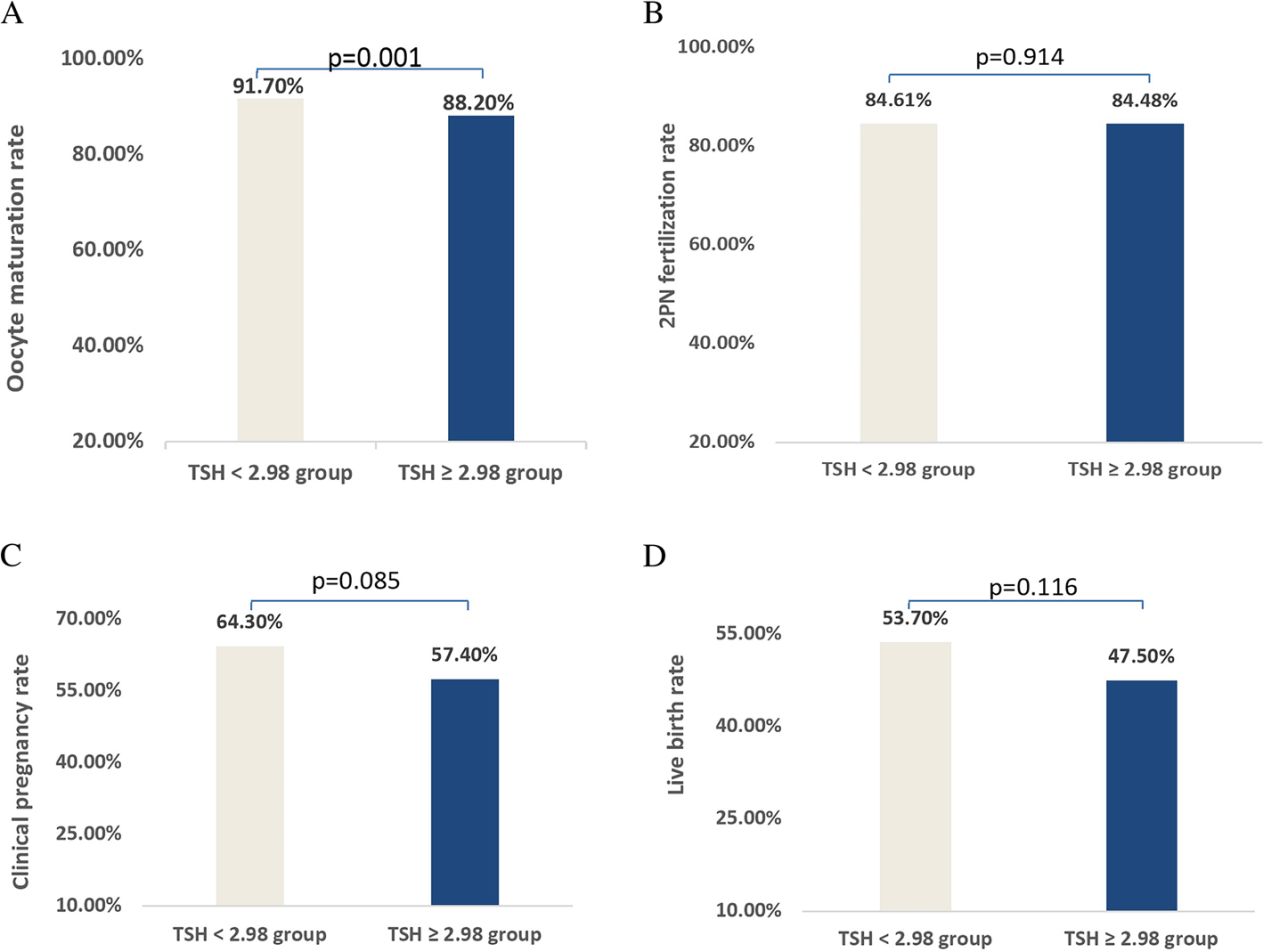
Distribution of Reproductive outcomes of TSH < 2.98 group and TSH ≥ 2.98 group. **A** Oocyte maturation rate; **B** 2PN fertilization rate; **C** Clinical pregnancy rate; **D** Live birth rate

Five hundred ninety-five PCOS patients were divided into two groups according to the threshold of TSH 2.98 in ROC curve. The results showed that the oocyte maturation of TSH < 2.98 group was significantly lower than that of TSH ≥ 2.98 group (91.7% vs 88.2%, *P* = 0.001) ( Fig. 1A); There was no difference in 2PN fertilization rate between the two groups (84.61% vs 84.48%, *P* = 0.914) ( Fig. 1B); The clinical pregnancy rate (64.3% vs 57.4%, *P* = 0.085) ( Fig. 1C)and live birth rate (53.7% vs 47.5%, *P* = 0.116) ( Fig. 1D)in TSH < 2.98 group were lower than those in TSH ≥ 2.98 group, but the difference was not statistically significant.

## Discussion

Thyroid hormone is an endocrine hormone that can maintain nervous system excitation, promote the development of tissues and organs, and regulate metabolism [12].

Due to the interaction between the hypothalamus pituitary ovary axis and the hypothalamus pituitary thyroid axis, thyroid hormone can have negative feedback effect on hypothalamus and pituitary. When TSH level is abnormal, it will affect hypothalamus function, resulting in abnormal hypothalamus pituitary gonad axis function [5–7]. Some studies have shown that the majority of female patients with thyroid dysfunction have symptoms such as irregular menstrual cycle, rare menstruation, irregular uterine bleeding, and some of them have polycystic ovarian changes with abnormal blood glucose metabolism and lipid metabolism disorder [19–21], which are considered to be related to insulin resistance and islet cell damage. At the same time, the imbalance of gonadal axis in PCOS patients may affect the pituitary thyroid axis. Many studies found that PCOS patients with severe hyperandrogenemia and insulin resistance are prone to be associated with abnormal thyroid function [22, 23]. Compared with normal women of childbearing age, PCOS patients are often accompanied by elevated TSH [8–10]. It is not known whether the elevated TSH levels in PCOS patients with normal thyroid function is associated with negative outcomes of in-vitro fertilization, such as poor oocyte maturation, low fertilization rate and high incidence of OHSS [24–26].

Our study showed that the average TSH level of PCOS patients with normal thyroid function was significantly higher than that of non-PCOS patients (2.42 ± 0.86VS2.00 ± 0.89, *P* < 0.001). This is consistent with the results of literature studies reported so far. The oocyte maturation rate of PCOS group was lower than that of non-PCOS group (90.9% vs 92.4%, *P* = 0.02), which further led to the lower 2PN fertilization rate in PCOS group than that in non-PCOS group (84.57% vs 86.77%, *P* = 0.02). However, there was no significant difference in cleavage rate and high-quality embryo rate between the two groups, and the pregnancy outcomes including clinical pregnancy rate, abortion rate and live birth rate were not significantly different between the two groups. We believe that this may be due to the development of IVF/ICSI therapy and the improvement of embryo culture system, which improves the adverse effects of elevated TSH on oocytes, so there was no significant difference between embryo outcome and pregnancy outcome. We adopt multi-factor linear regression analysis to evaluate the relationship between oocyte maturation and related variables. The results showed that TSH was negatively correlated with oocyte maturation of each patient [β = -124, *P* = 0.013,95% CI (- 0.027 ~ -0.003)], which suggested that TSH might be an independent risk factor for oocyte maturation in PCOS patients with normal thyroid function when undergoing IVF-ET.

TSH receptors are widely expressed in human oocytes, cumulus cells and granulosa cells of ovary. TSH cooperates with FSH to regulate the proliferation, apoptosis and morphological differentiation of granulosa cells, and play a direct role in promoting the function of granulosa cells [27]. TSH promotes the production of LH and hCG receptors on the surface of granulosa cells,while there is a close relationship between granulosa cells in follicles and oocytes, and gap junctions exist between them, the information transmission, mutual promotion and synchronous development between cells was put in to effect depend on the connexins [28].The functional status of granulosa cells directly affects the maturation of oocytes, granulosa cells also support oocyte maturation in an endocrine manner, and affects fertilization and even embryo development potential. Those maybe the main mechanism of the influence of TSH on oocyte quality in PCOS patients. In addition, the E2 levels on hCG trigger day of PCOS group is greater than that of non-PCOS group, and it is reported that the supraphysiological E2 levels during COH will also lead to the transient increase of TSH, this is mainly due to the increase of thyroxine binding protein caused by the super physiological high estrogen level in the serum, which reduces the concentration of free thyroid hormone and then causes the increase of serum TSH. Studies shown that the concentration of TSH increases with the increase of super physiological E2 level [29]. This may be another mechanism by which TSH affects oocyte maturation rate in PCOS group. However, the impact of of COH on TSH may be transient, the increased TSH after COH can not really reflect the state of thyroid function. TSH level after COH was not detected in this study, which is the deficiency of this paper.

There is no unified standard for what levels TSH should be controlled before IVF-ET at present. The American Society for reproductive medicine (ASRM) issued guidelines which suggested that if the TSH level of women of childbearing age before pregnancy is higher than 2.5mIU/ L, it is feasible to monitor the serum TSH level for many times. Once the serum TSH level is higher than 4.0mIU/L, levothyroxine can be used to maintain the serum TSH level below 2.5mIU/ L [14]. But it is controversial whether or not to use 2.5mIU/L thresholds for upper limit of TSH in first-trimester pregnancy and infertility [30–32].

In this study, we plotted the ROC curve of the effect of TSH on the oocyte maturity of each PCOS patient. The results showed that the area under the curve was 0.550, *p* = 0.037, and the specificity was 28.9%, the sensitivity was 83.0%. According to the cut-off threshold TSH 2.98uIU/ml, the patients were divided into two groups. There was a significant difference in oocyte maturity between the two groups (*p* < 0.001); The clinical pregnancy rate (64.3% vs57.4%, *P* = 0.085) and Live birth rate (53.7% vs 47.5%, *P* = 0.116) in TSH < 2.98 group was higher than that of TSH ≥ 2.98 group, but the difference was not statistically significant. Therefore, our results suggest that when TSH ≥ 2.98uIU/ml, the possibility of oocyte immaturation is higher (specificity 28.9%, sensitivity 83.0%), and it may have a negative effect on pregnancy outcomes.

### Conclusion

The TSH level of PCOS patients with normal thyroid function is higher than that of normal people, and it is negatively related to the oocyte maturation in IVF. We suggest that for PCOS patients with normal thyroid function, when TSH is higher than 2.98uIU/ml, intervention should be given before IVF cycle was conducted, which may be helpful to improve oocyte maturation.

## Data Availability

Not applicable.
